# Cu_5_FeS_4_ Nanoparticles With Tunable Plasmon Resonances for Efficient Photothermal Therapy of Cancers

**DOI:** 10.3389/fbioe.2020.00021

**Published:** 2020-02-18

**Authors:** Lei Yuan, Weiwei Hu, Hui Zhang, Long Chen, Jianyu Wang, Qiang Wang

**Affiliations:** ^1^Xuzhou Cancer Hospital, Xuzhou, China; ^2^The Affiliated Hospital of Xuzhou Medical University, Xuzhou, China

**Keywords:** Cu_5_FeS_4_ nanoparticles, plasmon resonances, bimetal copper-based chalcogenides, photothermal agents, photothermal therapy

## Abstract

Localized surface plasmon resonances (LSPRs) in heavily doped copper chalcogenides are unique because LSPR energy can be adjusted by adjusting doping or stoichiometry. However, there are few investigations on the LSPRs of bimetal copper-based chalcogenides. Herein, bimetal Cu_5_FeS_4_ (CFS) nanoparticles were synthesized by a facile hot injection of a molecular precursor. The tunable plasmon resonance absorption of CFS nanoparticles is achieved by the decrease of the ratio of copper to iron and the treatment of n-dodecylphosphoric acid (DDPA). After surface modification with polyethylene glycol (PEG), the CFS nanoparticles with a plasmon resonance absorption peak at 764 nm can serve as promising photothermal agents, showing good biocompatibility and excellent photothermal performance with a photothermal conversion efficiency of up to 50.5%, and are thus used for photothermal therapy of cancers under the irradiation of an 808-nm laser. Our work provides insight into bimetal copper-based chalcogenides to achieve tunable LSPRs, which opens up the possibility of rationally designing plasmonic bimetal copper-based chalcogenides.

## Introduction

Localized surface plasmon resonances (LSPRs) of nanostructures contribute to enhanced tunable optical absorption in the near-infrared (NIR) region, which is great for the improvement of the photothermal performance of photothermal nanoagents (Luther et al., 2011; Manthiram and Alivisatos, 2012; Yang et al., 2013; Li et al., 2015). As noble metals are stable under various conditions and have high carrier densities, most reports of LSPRs have been performed on them (Huang et al., 2011a,b; Manthiram and Alivisatos, 2012). There are also reports of LSPRs in doped semiconductor nanostructures, including copper chalcogenides (Liu et al., 2013; Balitskii et al., 2014), tin oxides (Kanehara et al., 2009; Garcia et al., 2011), and zinc oxide (Buonsanti et al., 2011). LSPRs in heavily doped copper chalcogenides are unique because LSPR energy can be adjusted by adjusting doping or stoichiometry (Luther et al., 2011; Tian et al., 2011; Li et al., 2015). There have been a few reports on copper chalcogenides with enhanced photothermal performance by adjusting doping for photothermal therapy of cancers (Hessel et al., 2011; Tian et al., 2013; Li et al., 2014a, 2015).

The LSPRs of copper-based chalcogenide compounds is an important factor affecting their photothermal conversion efficiency. As the plasmon resonance absorption of gold nanostructures is much higher than the inter-band absorption of organic materials, the photothermal conversion efficiency of gold nanostructures is one of the currently reported high photothermal conversion materials (Yavuz et al., 2009; Tang et al., 2012). The hole-doped copper-based chalcogenides have a high extinction coefficient similar to that of the metal plasmon resonance absorption. Tian et al. developed a novel hole-doped semiconductor photothermal conversion material, i.e., Cu_9_S_5_ disk-shaped nanocrystals (Tian et al., 2011). The extinction coefficient and photothermal conversion efficiency of this Cu_9_S_5_ nanocrystal at 980 nm are as high as ~1.2 × 10^9^ M^−1^ cm^−1^ and 25.7%, respectively, which are higher than the extinction coefficient (~1.1 × 10^9^ M^−1^ cm^−1^) and the photothermal conversion efficiency (23.7%) of gold nanorods under the same test conditions. Therefore, the photothermal conversion efficiency of the hole-doped semiconductor can be improved by regulating the plasmon resonance absorption. Zhao et al. and Luther et al. have demonstrated that the plasmon resonance absorption of hole-doped copper-based chalcogenides depends on the carrier concentration in the doped semiconductor (Zhao et al., 2009; Luther et al., 2011). Therefore, the plasmon resonance absorption of hole-doped copper-based chalcogenide is different from that of metals. It can control the position and intensity of the plasmon resonance absorption peak not only by changing the morphology and particle size of the semiconductor, but also by changing its structure and composition. For a hole-doped alloy copper-based chalcogenide [such as Cu_2−x_S_*y*_Se_1−y_, Cu_2−x_S_*y*_Te_1−y_ (0 ≤ x, y ≤ 1)] compound, the bandgap can be controlled by tuning the ratio of chalcogen (S and Se, Te) (Wang et al., 2011; Liu et al., 2013), and it is also possible to increase the hole density by increasing the Cu defect to achieve the regulation of the position and intensity of local surface plasmon resonance absorption (Dorfs et al., 2011; Garcia et al., 2011; Hsu et al., 2011; Scotognella et al., 2011). Wang et al. have prepared Cu_2−x_S_*y*_Se_1−y_ compounds by a simple one-step synthesis method and have realized the effective regulation of plasmon resonance absorption by regulating the composition and structure of the compounds (Wang et al., 2011). Many groups have reported hole-doped plasma resonance absorption effect of copper chalcogenides. Recently, it has been reported that the doping of indium can change the particle size, morphology, and crystal phase of Cu_2−x_S nanocrystals, which in turn affects its plasmon resonance absorption (Wang and Swihart, 2015). In addition, the synthesized copper chalcogenide compounds can still achieve the regulation of plasma absorption by oxidation or reduction (Kriegel et al., 2012). Balitskii et al. have reported an interesting phenomenon: They synthesized oleylamine-coated Cu_2−x_Se nanocrystals, and then partially exchanged the oleylamine ligand with n-dodecylphosphoric acid (DDPA) or 1-dodecanethiol (DDT) to blue or red shift the plasma absorption of the Cu_2−x_Se nanocrystals (Balitskii et al., 2014). Therefore, plasma absorption can also be adjusted by changing the surface ligand. In summary, the plasmon resonance absorption of copper chalcogenides can be changed and improved by controlling the doping, morphology, structure, composition, particle size, and surface ligand of the hole-doped copper chalcogenide compounds, thereby improving the photothermal conversion efficiency. However, there are few investigations on the LSPRs of bimetal copper-based chalcogenides.

In this work, we report Cu_5_FeS_4_ (CFS) nanoparticles as a promising photothermal agent for efficient photothermal therapy of cancers. The plasmon resonance absorption of CFS nanoparticles could be tuned by the increase of the ratio of iron to copper during the reaction leading to the increased defect structure in CFS. The plasma absorption of CFS nanoparticles could also be tuned by exchanging the oleylamine ligand with n-dodecylphosphoric acid (DDPA). The CFS nanoparticles with a plasmon resonance absorption peak at 764 nm can be used as the 808-nm laser-driven photothermal agents for the photothermal therapy of cancers after surface modification by polyethylene glycol (PEG). To the best of our knowledge, this work is the first report on the tunable plasmon resonance of CFS.

## Materials and Methods

### Synthesis of Molecular Precursor

CuCl_2_·2H_2_O and FeCl_3_·6H_2_O were fully dissolved in water, and then a solution of sodium diethyldithiocarbamate (SDEDTC) was added and stirred for 1 h. The brown products were then filtered, washed with water several times, and then dried at room temperature before use.

### Synthesis of CFS Nanoparticles

Twenty-five milliliters of oleylamine (Aladdin) was added in a flask, and then heated to 120°C within under the magnetic stirring with a continuous flow of dry argon gas. The solution was then heated to 300°C and kept for 30 min. Subsequently, another 5 ml of oleylamine containing the molecular precursors was injected into the above hot solution. The reaction was kept for 10 min. After cooling to room temperature, the end products were collected via centrifugation and washed with ethanol twice.

### DDPA Treatment

Three hundred microliters of the resulting solution containing about 4 mg of CFS nanoparticles was mixed with 4 ml of 0–20 mM DDPA in toluene. The solution was then sonicated for about 15 min.

### Surface Modification

Two milligrams of PEG-NH_2_ was added in 4 ml of the resulting solution containing about 10 mg of CFS nanoparticles in toluene. The resulting solution was then sonicated for 10 min. Thereafter, 10 ml of deionized water was added in the above solution and sonicated for another 10 min. The products were obtained by centrifugal collection and washed with ethanol twice.

### Characterization

TEM (JEOL JEM-2010F, Japan) was used to measure the morphology and size of the CFS nanoparticles. UV-Vis-NIR absorbance spectra of CFS nanoparticles were obtained through a UV-Visible spectrophotometer (UV-1900, Phenix) at room temperature. X-ray photoelectron spectra (XPS, ESCA-Lab 250Xi) were used to test the oxidation state analysis of CFS nanoparticles. XRD (Bruker D4) was used to measure the crystal phase of CFS nanoparticles. ICP-AES (Leeman Laboratories Prodigy) was used to measure the concentration of ions released from CFS nanoparticles.

### Photothermal Effect

To measure the photothermal effect of the CFS nanoparticles, CFS nanoparticles with different concentrations dispersed in deionized water were irradiated under an 808-nm laser (0.5 W cm^−2^) for 5 min. Temperature change of CFS nanoparticles was monitored and recorded by an infrared thermal camera.

### Cellular Experiment *in vitro*

K7M2 cells were used as a model to assess the cytotoxicity of the CFS nanoparticles to cancer cells. K7M2 cells were cultured in Roswell Park Memorial Institute (RPMI-1640) and incubated at 37°C in the presence of 5% CO_2_. For cytotoxicity evaluation of the CFS nanoparticles *in vitro*, K7M2 cells were seeded in the 96-well-plates at a density of 1 × 10^4^ cells every well. After cultivation for 24 h, the suspension medium was aspirated by pipette and washed with PBS three times. CFS nanoparticles dispersed in RPMI-1640 with different concentrations were added into the wells one by one. After co-culture 24 h, a standard CCK8 evaluation was used to investigate the cell viability of CFS nanoparticles to K7M2 cells. For photothermal therapy effect of K7M2 cells *in vitro* using CFS nanoparticles, K7M2 cells were cultured CFS nanoparticles in RPMI-1640 for 24 h. These cells were then irradiated upon an 808-nm laser at a power density of 0.5 W cm^−2^ for 5 min. After then, the K7M2 cells were stained with both calcein AM and PI and then imaged by a Leica DMi8 fluorescence microscope. All of the tests were measured three times.

### Photothermal Therapy *in vivo*

The immunodeficiency nude mice were subcutaneously injected by K7M2 cells (4 × 10^6^ cells for each mouse) into the left thigh to obtain the desired tumor model. K7M2 model mice were then divided randomly into four groups (six mice for each group) when the tumor volumes reached about ~100 mm^3^. PBS solution containing CFS nanoparticles (50 ppm, 100 μl) or PBS solution alone was injected intravenously into the tumor sites of the nude mice. The mice with or without the CFS nanoparticle's injection were then irradiated to an 808-nm laser with a power density at 0.5 W cm^−2^ for 5 min. During the treatment, the temperature changes of tumor surface were monitored and recorded by a NIR thermal medical camera. After the indicated treatments, we recorded the body weight and tumor size of the mice in four groups every 2 days to evaluate the photothermal therapy effect. Relative tumor volumes were calculated as *V*/*V*_0_, where *V*_0_ was the initial tumor volume before the therapy.

### Long-Term Toxicity Analysis

As for the tumors' histological examination analysis, a mouse from the each group was sacrificed under anesthesia after the indicated treatment. Then, the tumors from the sacrificed mice were harvested, sectioned into 4-μm slices, and stained with hematoxylin and eosin (H&E). The slices were examined via a microscope. Mice were sacrificed to collect blood sample for blood biochemistry and complete blood panel analysis after intervals of 3, 6, 9, and 12 days, respectively.

## Results and Discussion

**Figure 1:**
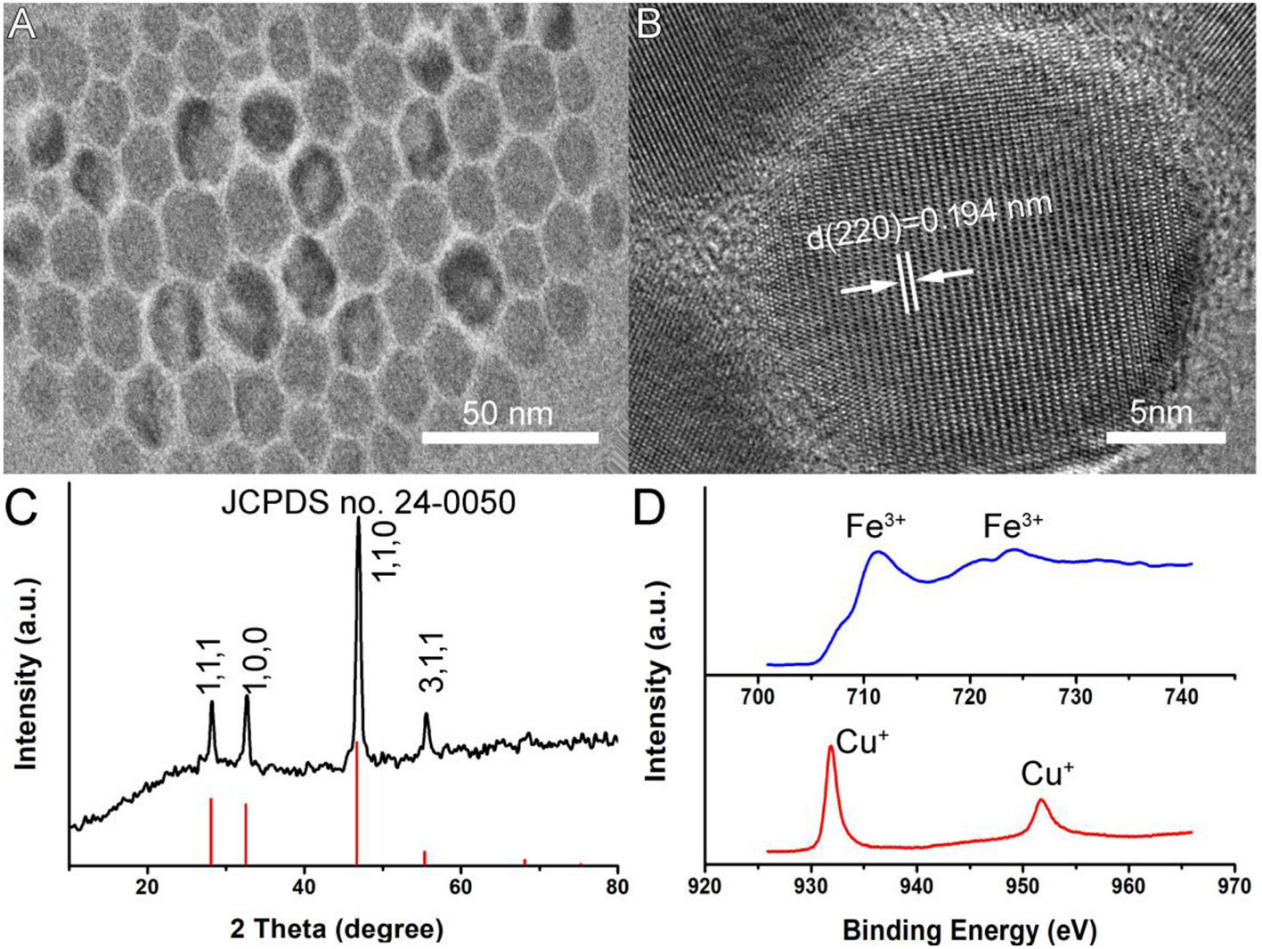
**(A)** A typical TEM image of CFS nanoparticles. **(B)** The high-resolution TEM image of CFS nanoparticles. **(C)** XRD patterns of CFS nanoparticles. **(D)** The high-resolution XPS spectra of Fe 2p (blue line) and Cu 2p (red line) in CFS nanoparticles.

Oleylamine-capped CFS nanoparticles were synthesized by a facile hot injection of a molecular precursor. The molecular precursors were obtained by reacting CuCl_2_·2H_2_O as well as a certain molar ratio of FeCl_3_·6H_2_O with sodium diethyldithiocarbamate (SDEDTC). Figure 1A presents the transmission electron microscopy (TEM) image of CFS nanoparticles obtained from the reaction with a ratio of 20% for iron source to copper source. One can see that the as-prepared CFS products showed very good dispersion from the TEM image. The size of CFS nanoparticles was found to be 18.2 nm based on the statistics of 100 nanoparticles from the TEM images (Figure S1). Figure 1B showed more microstructure information from the high-resolution TEM; an interplanar spacing of 0.194 nm was observed, assigned to (110) planes of bornite structured Cu_5_FeS_4_. In addition, selected area electron diffraction of the individual CFS nanoparticles from the high-resolution TEM can be indexed to the [110] zone axis of bornite structured Cu_5_FeS_4_, which indicated the single-crystalline nature of these nanoparticles (Figure S2). As shown in Figure 1C, all of the main peaks of X-ray XRD pattern of the CFS products can be matched well with those of bornite structured Cu_5_FeS_4_ (JCPDS file no. 24-0050), showing a high crystallinity for pure CFS. XPS results (Figure S3) demonstrated that the obtained products mainly contain Cu, Fe, and S elements. We also analyzed the valency state of Cu and Fe in CFS nanoparticles. In Figure 1D, it exhibited the high-resolution Cu 2p spectrum (red line) and Fe 2p spectrum (blue line) for the CFS nanoparticles. The binding energy peaks at 931.8 and 951.7 eV could belong to Cu (I) coordinated to Cu in CFS nanoparticles (Chang et al., 2013). Generally speaking, the existence of Cu(II) in copper-based photothermal agents could contribute to the defect structure, thus resulting in NIR absorption (Li et al., 2015). However, no binding energy peak at around 940 eV was detected, indicating that there was no Cu (II) existing in CFS nanoparticles (Chang et al., 2013). Therefore, no copper defect structure was found in CFS obtained from the reaction with a ratio of 20% for iron source to copper source. In addition, the binding energy peaks at 711.2 and 724.1 eV could be assigned to Fe(III) coordinated to Fe in CFS nanoparticles (Guan et al., 2018). Based on the obtained results, we can conclude that CFS nanoparticles with high purity and crystallinity were achieved.

**Figure 2:**
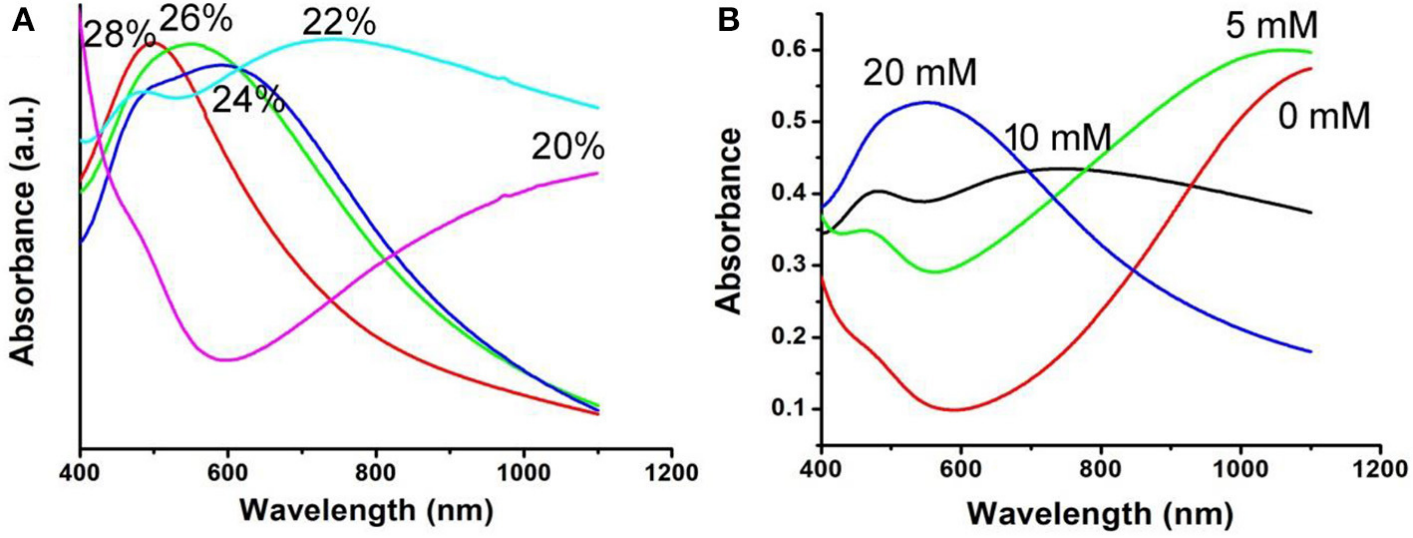
**(A)** UV-vis-NIR spectra of the CFS nanoparticles obtained from the addition of precursors with different ratios for iron source to copper source. **(B)** UV-vis-NIR spectra of the CFS nanoparticles after the treatment of DDPA.

The optical absorption spectrum of CFS nanoparticles, obtained from the reaction with a ratio of 20% for iron source to copper source, showed a peak around 1,100 nm. The NIR absorption may be attributed to 3d electronic transitions from the valence band to an intermediate band, similar to CuFeS_2_ nanocrystals (Ghosh et al., 2016) and CuCo_2_S_4_ nanocrystals (Li et al., 2017). The ratio of Cu to Fe was found to be 4.99, confirmed by ICP-AES. Interestingly, the ratio of 4.82, 4.75, 4.71, and 4.67 for Cu to Fe can also be obtained by the addition of precursors with the ratio of 22, 24, 26, and 28% for iron source to copper source, respectively, indicating different copper deficiencies. The high-resolution XPS spectra of Fe 2p and Cu 2p in CFS nanoparticles obtained from the reaction with a ratio of 28% for iron source to copper source are given in Figure S4. It was found that the peaks of Cu^2+^ and Fe^2+^ were detected, further indicating the formation of defect structures (Chang et al., 2013; Guan et al., 2018). Previous work has demonstrated that the plasmon resonance absorption peak will shift blue as the copper defects increase (Balitskii et al., 2014). In order to verify it, we measured the optical absorption spectrum of CFS nanoparticles with different ratios of Cu to Fe. As expected, there was a blue shift with the increase of copper deficiencies (Figure 2A). It has been revealed that copper deficiency could be achieved by oxidizing copper in the crystal. When the CFS was prepared from the reaction with a ratio of 20% for iron source to copper source, the obtained CFS products were processed with DDPA to achieve CFS with different deficiencies. When treating the CFS nanoparticle solution with DDPA, a blue shift was observed (Figure 2B). The blue shift up to 500 nm was achieved when the concentration of DDPA was increased to 20 mM. After the treatment by 20 mM, the CFS nanoparticles showed little change, which can be confirmed from the TEM image and XRD pattern of CFS nanoparticles (Figure S5). Therefore, the LSPRs of CFS nanoparticles can be tuned by the addition of precursors with different ratios for iron source to copper source and the treatment of DDPA, respectively.

**Figure 3:**
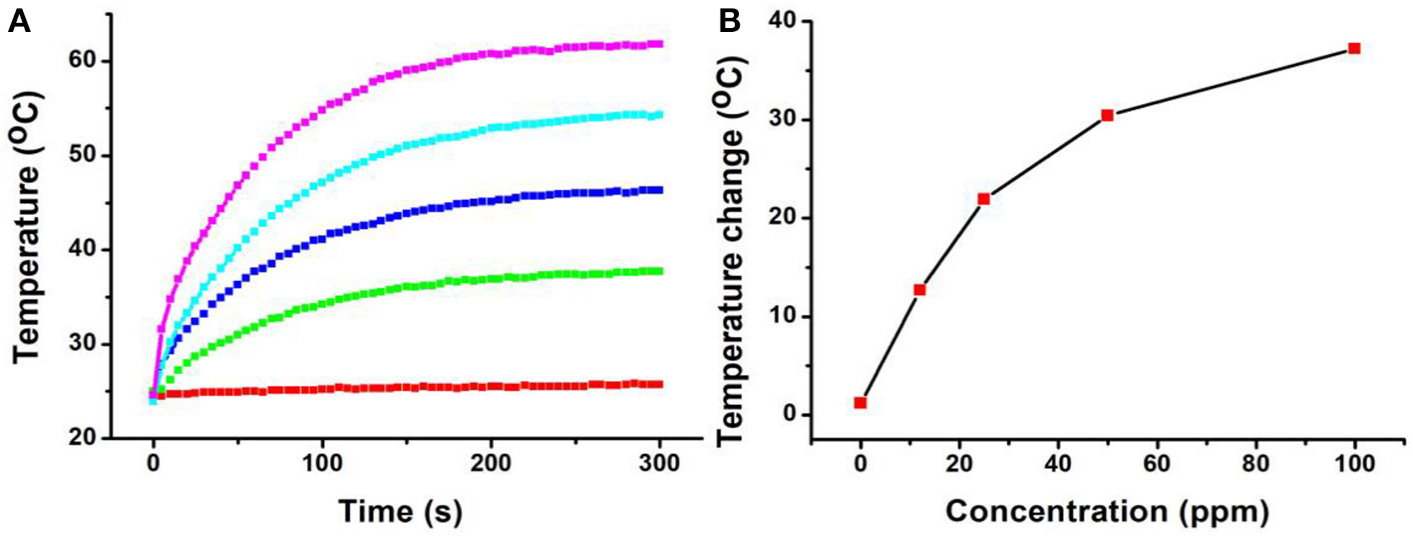
**(A)** The temperature changes of the CFS nanoparticles dispersed in water with different concentrations of Cu^2+^ (0–100 ppm) vs. the irradiation time of the 808-nm laser. **(B)** Relationship curves of temperature change as a function of the concentration of CFS nanoparticles.

In general, both 980-nm light and 808-nm light have been used as laser sources for the photo-responsive imaging and treatments of tumors due to their security and deep tissue penetration (Smith et al., 2009). However, as the main constituent of living organisms, the absorption intensity of the water at 980 nm is confirmed to be 30 times higher than that at 808 nm (Li et al., 2014b). It has been proved that a 980-nm laser irradiation at a power intensity of 0.72 W/cm^2^ can make the temperature of the pure water increase by 15.1°C, while only 3.0°C for an 808-nm laser in the same conditions (Li et al., 2014b). This means that the 808-nm laser exhibits deeper tissue penetration and less photo-damage to the surrounding healthy tissues compared to the 980-nm laser (Wu and Butt, 2016). In our experiments, The CFS nanoparticles, obtained from the reaction with a ratio of 22% for iron source to copper source, showed a plasmon resonance absorption peak at 764 nm, which is close to 808 nm. Therefore, an 808-nm laser was used as the excitation source for photothermal therapy. Photothermal agents should be hydrophilic before realizing their biological application; thus, a ligand exchange process was performed to modify the CFS nanoparticles' surface property using PEG. We then measured the photothermal effect of PEG-coated CFS nanoparticles. As shown in Figure 3, CFS nanoparticles exhibited a concentration-dependent photothermal effect. As a control, the pure water showed almost no temperature raise under the exposure of an 808-nm laser (0.5 W cm^−2^). However, with the increase of the concentration of CFS nanoparticles (i.e., 12, 25, 50, and 100 ppm), the temperature was increased by 12.7–37.2°C, indicating that CFS nanoparticles can efficiently convert NIR light energy to heat energy.

**Figure 4:**
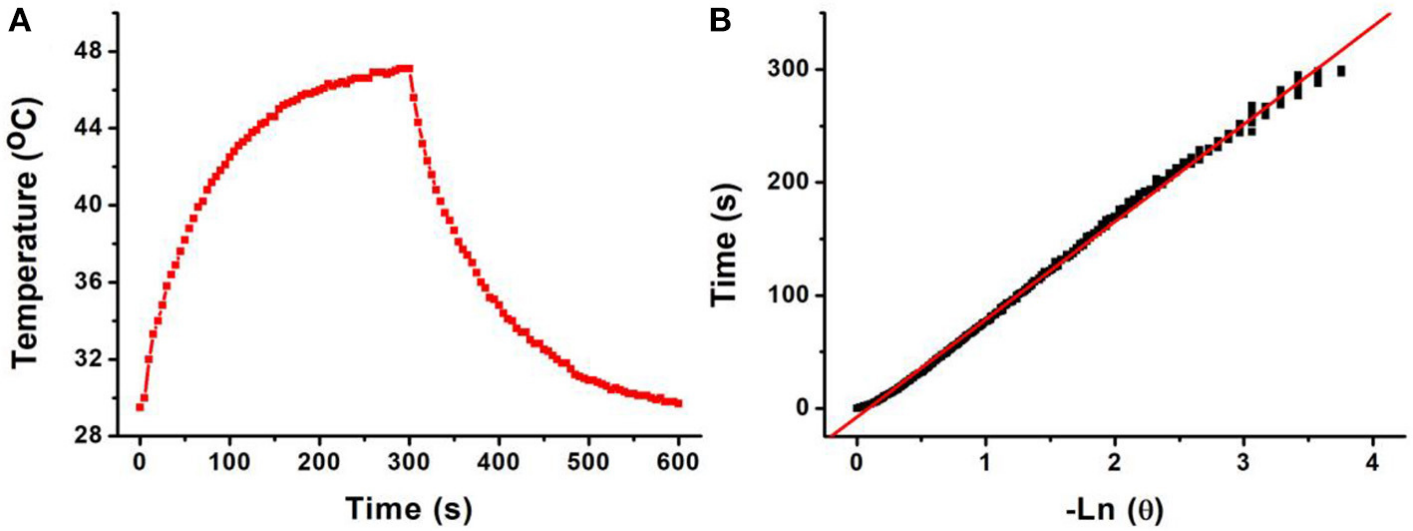
**(A)** Photothermal effect of CFS nanoparticles under the irradiation of an 808-nm laser for 300 s; then, the laser was shut off and the temperature was recorded for another 300 s. **(B)** Time constant of CFS NCs from the system. τ_s_ = 73.2 s.

The 808-nm laser-driven photothermal conversion efficiency of the CFS nanoparticles was also measured and calculated by Roper et al.'s reported method (Roper et al., 2007). The CFS nanoparticles with a concentration of 50 ppm were exposed to the 808-nm laser (0.192 W) for 300 s. The laser was shut off when the temperature of the system reached a steady state (Figure 4A). Cooling time constant was measured to confirm system's rate of heat transfer (Figure 4B). Then, the photothermal conversion efficiency (η_*T*_) of CFS nanoparticles could be calculated by the following:

(1) $$\begin{array}{l} \eta_{T}=\frac{Q_{1}-Q_{0}}{I\left(1-10^{A_{\lambda }}\right)} \end{array}$$

In which *Q*_1_ and *Q*_0_ are the rate of heat input (in units of mW) of the solvent with and without CFS nanoparticles. *I* is the laser power (in units of mW, 192 mW). *A*_λ_ is the absorbance (0.8345) at irradiation wavelength (808 nm). The value of *Q*_1_ and *Q*_0_ are derived according to:

(2) $$\begin{array}{l} Q=hA\left(T_{\max}-T_{amb}\right) \end{array}$$

*h* is the heat transfer coefficient, and *A* is the container's surface area. *T*_amb_ is the ambient surrounding temperature, *T*_max_ is the maximum temperature of the system, and (*T*_max_ – *T*_amb_) was 17.6°C obtained from Figure 4A. The *Q*_0_ was independently measured with a quartz cuvette cell containing pure water and calculated to be 18.7 mW. The value of hA can be calculated from:

(3) $$\begin{array}{l} hA=\frac{m_{D}c_{D}}{\tau_{s}} \end{array}$$

where *m*_D_ and *c*_D_ are the mass (0.1 g) and heat capacity (4.2 J g^−1^) of pure water, which was used as solvent, respectively. τ_s_ is the sample system time constant. According to the achieved data, the 808-nm laser-driven photothermal conversion efficiency (η_*T*_) of the CFS nanoparticles was calculated to be 50.5%, indicating the promising potential for photothermal therapy of cancers. To evaluate the NIR photostability of the CFS nanoparticles, the aqueous dispersion (50 ppm) was irradiated with 808-nm laser (1.0 W cm^−2^) for 10 min (LASER ON, Figure S6), respectively, followed by naturally cooling to room temperature for 30 min (without irradiation, LASER OFF). It was shown that there was little loss of the maximum temperature elevation after four cycles of LASER ON/OFF. The result indicated that the CFS nanoparticles showed good NIR photostability.

**Figure 5:**
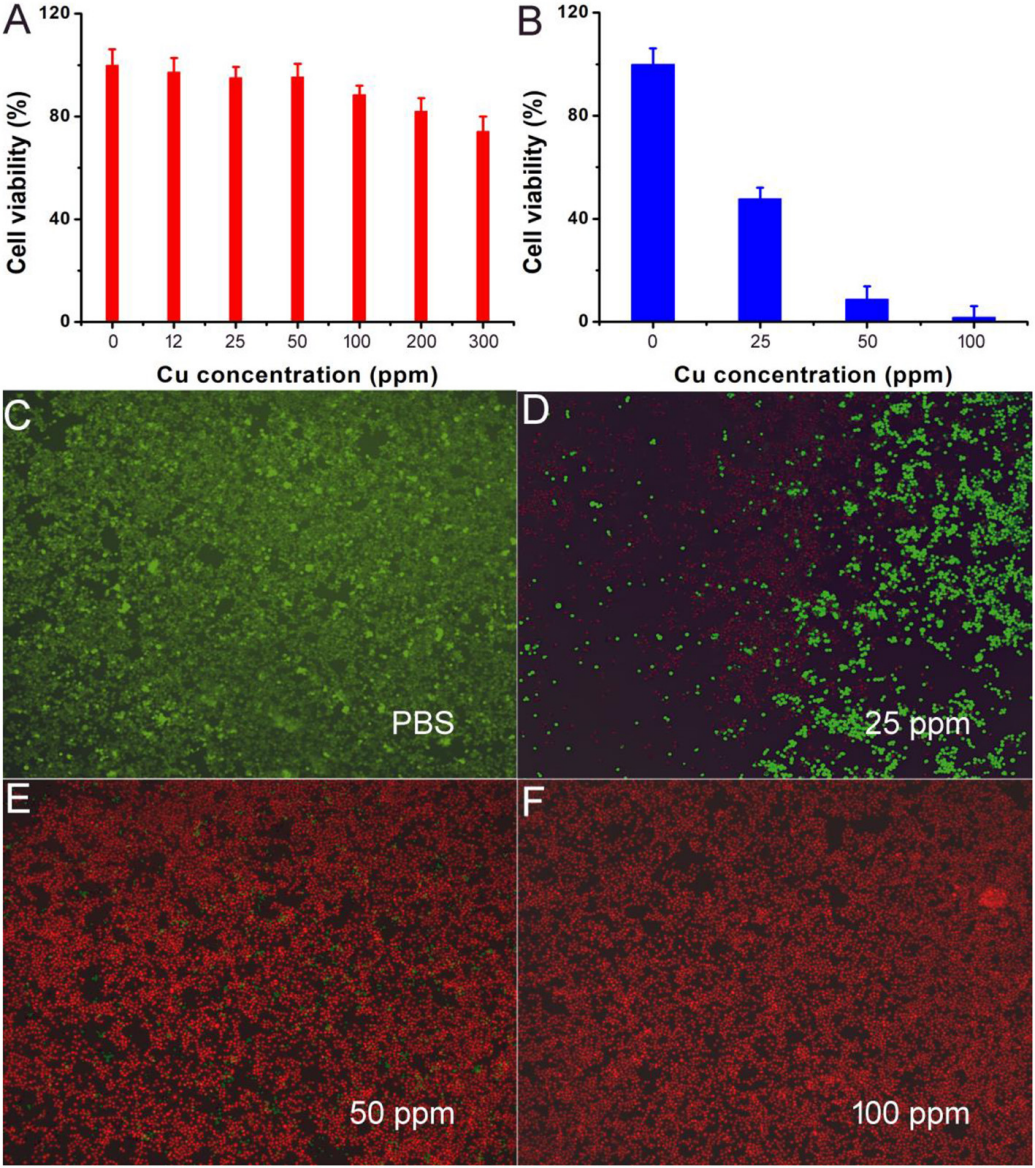
**(A)** Cell viability incubated with different concentrations of CFS nanoparticles. **(B)** Cell viability after the indicated photothermal treatments. **(C–F)** Live/dead cell staining analysis after photothermal therapy with CFS nanoparticles at different concentrations. Magnification: 100 times.

Motivated by the excellent photothermal performance of CFS nanoparticles, we evaluated the potential of these nanoparticles as photothermal agents. Before realization of their biological application, the cytotoxicity of CFS nanoparticles was evaluated by a standard CCK-8 assay method with K7M2 cells (Figure 5A). It can be seen that these CFS nanoparticles appeared to be very low toxicity. With a concentration up to 200 ppm, the cell viability can still be up 80%. The *in vitro* photothermal therapy to K7M2 cells using CFS nanoparticles was then studied. After the treatment with varied concentration of CFS nanoparticles, a standard CCK-8 evaluation was used to test the cell viability (Figure 5B). The cell mortality rate increased with the increase of the concentration. The cell mortality rate of the treatment combined CFS nanoparticles (50 ppm) and the irradiation of an 808-nm laser was ~92%, demonstrating an excellent photothermal effect *in vitro*. When the concentration of nanoparticles was increased to 100 ppm, the cell death rate was increased by 7%. Thus, the optimized concentration should be 50 ppm. In addition, to visualize the *in vitro* photothermal therapy effect of CFS nanoparticles, K7M2 cells after the showing treatments were co-stained with calcein-AM and propidium iodide. The results (Figures 5C–F) were consistent with the result of the CCK-8 assay, demonstrating the efficient photothermal effect *in vitro*.

**Figure 6:**
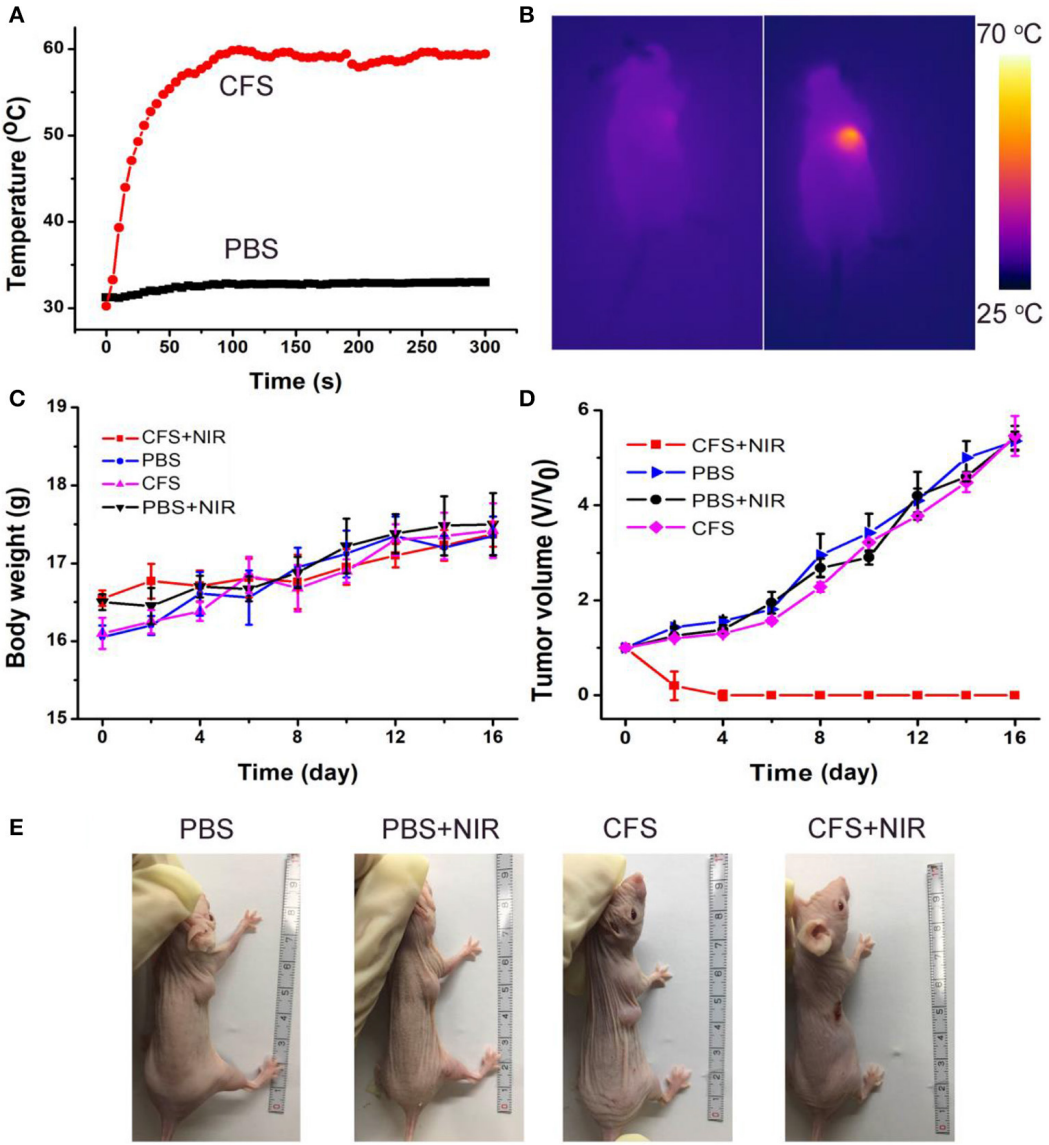
**(A)** Temperature curves of tumors as a function of irradiation time of an 808-nm laser. **(B)** The infrared thermal images of the tumor-bearing mice intravenously injected with the PBS (the left mouse) or CFS nanoparticles (the right mouse), respectively. **(C)** Body weight curves over time in the four groups after indicated treatments. **(D)** Growth curves of tumors over time in the four groups after indicated treatments. **(E)** Photographs of mice in the four groups after the indicated photothermal treatments for 14 days.

The photothermal therapy effect of cancers *in vivo* was evaluated. The tumor-bearing mice were divided randomly into four groups: (a) CFS nanoparticle injection + 808-nm laser irradiation (CFS+NIR); (b) PBS + 808-nm laser irradiation (PBS+NIR); (c) PBS injection (PBS); and (d) CFS nanoparticle (CFS). The temperatures of tumor surface injected with CFS nanoparticles could dramatically increase from ~30 to ~59°C, resulting from the photothermal effect of CFS nanoparticles (Figure 6A). As a control, the temperatures of tumor surface injected with PBS solution only increased by less than 3°C. During the treatment, a medical thermal camera was used to monitor and record the temperature change of tumor sites. As expected, infrared thermal images with a high contrast were achieved (Figure 6B), indicating that CFS nanoparticles still showed excellent photothermal effect *in vivo*.

After the indicated treatment, the body weights and tumor sizes from each group were recorded every 2 days. We can see that there was almost no difference in body weight among the four groups of mice (Figure 6C), indicating the low toxicity of CFS at the given conditions. In addition, tumors of mice in group (a) disappeared and there was no reoccurrence observed (Figures 6D,E), while the tumors gradually increased and showed no difference in the three control groups. H&E staining analysis was also analyzed immediately after treatment to evaluate the photothermal therapy effect after the CFS/NIR laser treatment. The tumor cells in the three control groups showed very little change in morphology and size, while those in the experimental group (CFS+NIR) showed obvious necrosis, such as nuclear condensation, shrinkage of the malignant cells, lysis, and fragmentation (Figure S7). Therefore, CFS nanoparticles showed great potential for photothermal therapy of cancers.

**Figure 7:**
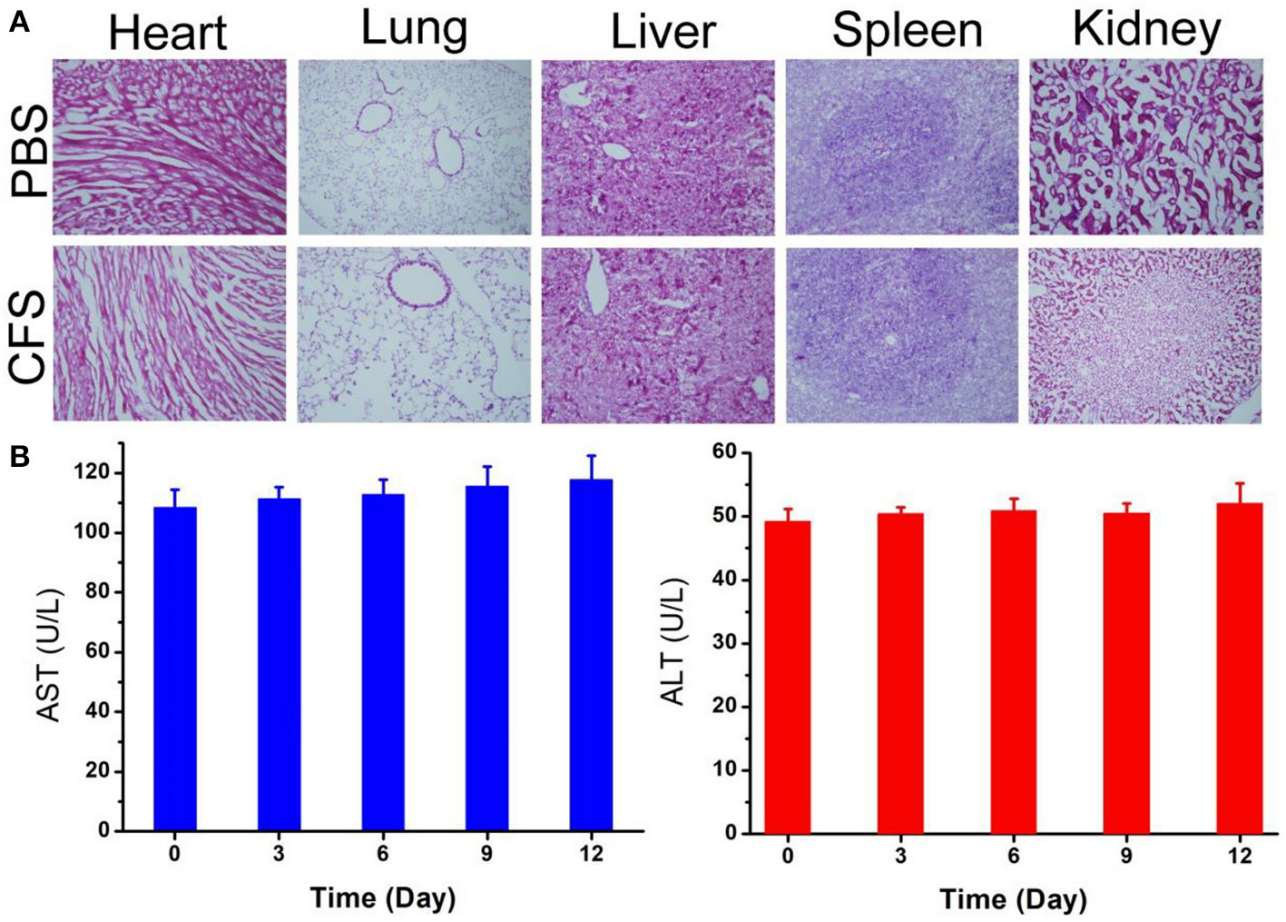
**(A)** H&E-stained slices of main organs. Magnification: 100 times. **(B)** Blood biochemistry of mice receiving intravenous injection of CFS at different time points. The examined parameters include aspartate aminotransferase (AST, left) and alanine aminotransferase (ALT, right).

As *in vivo* biosafety of nanomedicines is always of great concern for application in photothermal therapy, further bio-safety experiment on histological examination analysis with H&E staining for the main organs was conducted to observe the size, shape, and number of cells after the intravenous injection of CFS nanoparticles (10 mg/kg). From the HE staining of the major organs, including heart, kidney, spleen, liver, and lung, no inflammation or damage is observed (Figure 7A). The parameters related to the serum biochemistry (Figure 7B) showed no meaningful changes. The evidence further confirmed that the CFS nanoparticles have promising potential for photothermal therapy. However, deep systematic studies of pharmacokinetics and pharmacodynamics are still pretty important for future clinical application of such a material. This kind of material may also have other specific potential, such as magnetic resonance imaging, that can further transform the platform for multiple use to promote its clinical translation.

## Conclusion

In conclusion, hydrophilic Cu_5_FeS_4_ nanoparticles with a plasmon resonance absorption peak at 764 nm that served as an efficient photothermal agent have been successfully prepared by a facile thermal decomposition route and, subsequently, a surface modification process. The plasmon resonance absorption of Cu_5_FeS_4_ nanoparticles could be tuned by the decrease of the ratio of copper to iron and the treatment of DDPA leading to the formation of defect structures. Moreover, CFS nanoparticles showed excellent biocompatibility, demonstrated by the *in vitro* and *in vivo* toxicity results. With the exposure of the NIR light, the Cu_5_FeS_4_ nanoparticles can be used for efficient photothermal therapy of cancers.

## Data Availability Statement

All datasets generated for this study are included in the article/Supplementary Material.

## Ethics Statement

This study was carried out in accordance with the principles of the Basel Declaration and recommendations of the Institutional Animal Care and Use Committee of the Affiliated Hospital of Xuzhou Medical University. The protocol was approved by the Affiliated Hospital of Xuzhou Medical University.

## Author Contributions

LY and QW designed the project. LY, WH, and HZ carried out the experiment and performed the experimental data analysis. LY, LC, and JW wrote the paper. All authors contributed to discussion of the results.

### Conflict of Interest

The authors declare that the research was conducted in the absence of any commercial or financial relationships that could be construed as a potential conflict of interest.

## References

[B1] Balitskii O. A. Sytnyk M. Stangl J. Primetzhofer D. Groiss H. Heiss W. (2014). Tuning the localized surface plasmon resonance in Cu(2-x)Se nanocrystals by postsynthetic ligand exchange. ACS Appl. Mater. Interfaces 6, 17770–17775. 10.1021/am504296y 25233007 PMC4207552

[B2] Buonsanti R. Llordes A. Aloni S. Helms B. A. Milliron D. J. (2011). Tunable infrared absorption and visible transparency of colloidal aluminum-doped zinc oxide nanocrystals. Nano Lett. 11, 4706–4710. 10.1021/nl203030f 21970407

[B3] Chang J. Y. Lin J. M. Su L. F. Chang C. F. (2013). Improved performance of CuInS_2_ quantum dot-sensitized solar cells based on a multilayered architecture. ACS Appl. Mater. Interfaces 5, 8740–8752. 10.1021/am402547e 23937511

[B4] Dorfs D. Hartling T. Miszta K. Bigall N. C. Kim M. R. Genovese A. (2011). Reversible tunability of the near-infrared valence band plasmon resonance in Cu_2_-_*x*_Se nanocrystals. J. Am. Chem. Soc. 133, 11175–11180. 10.1021/ja2016284 21728384

[B5] Garcia G. Buonsanti R. Runnerstrom E. L. Mendelsberg R. J. Llordes A. Anders A.. (2011). Dynamically modulating the surface plasmon resonance of doped semiconductor nanocrystals. Nano Lett. 11, 4415–4420. 10.1021/nl202597n 21859093

[B6] Ghosh S. Avellini T. Petrelli A. Kriegel I. Gaspari R. Almeida G.. (2016). Colloidal CuFeS_2_ nanocrystals: intermediate fe d-band leads to high photothermal conversion efficiency. Chem. Mater. 28, 4848–4858. 10.1021/acs.chemmater.6b02192 29033496 PMC5634747

[B7] Guan G. Wang X. Li B. Zhang W. Cui Z. Lu X.. (2018). “Transformed” Fe3S4 tetragonal nanosheets: a high-efficiency and body-clearable agent for magnetic resonance imaging guided photothermal and chemodynamic synergistic therapy. Nanoscale 10, 17902–17911. 10.1039/c8nr06507a 30226246

[B8] Hessel C. M. Pattani V. P. Rasch M. Panthani M. G. Koo B. Tunnell J. W.. (2011). Copper selenide nanocrystals for photothermal therapy. Nano Lett. 11, 2560–2566. 10.1021/nl201400z 21553924 PMC3111000

[B9] Hsu S. W. On K. Tao A. R. (2011). Localized surface plasmon resonances of anisotropic semiconductor nanocrystals. J. Am. Chem. Soc. 133, 19072–19075. 10.1021/ja2089876 22044349

[B10] Huang X. Tang S. Liu B. Ren B. Zheng N. (2011a). Enhancing the photothermal stability of plasmonic metal nanoplates by a core-shell architecture. Adv. Mater. Weinheim. 23, 3420–3425. 10.1002/adma.201100905 21688329

[B11] Huang X. Tang S. Mu X. Dai Y. Chen G. Zhou Z.. (2011b). Freestanding palladium nanosheets with plasmonic and catalytic properties. Nat. Nanotechnol. 6, 28–32. 10.1038/nnano.2010.235 21131956

[B12] Kanehara M. Koike H. Yoshinaga T. Teranishi T. (2009). Indium tin oxide nanoparticles with compositionally tunable surface plasmon resonance frequencies in the near-IR region. J. Am. Chem. Soc. 131, 17736–17737. 10.1021/ja9064415 19921844

[B13] Kriegel I. Jiang C. Rodríguez-Fernández J. Schaller R. D. Talapin D. V. da Como E.. (2012). Tuning the excitonic and plasmonic properties of copper chalcogenide nanocrystals. J. Am. Chem. Soc. 134, 1583–1590. 10.1021/ja207798q 22148506

[B14] Li B. Wang Q. Zou R. Liu X. Xu K. Li W.. (2014a). Cu_7.2_S_4_ nanocrystals: a novel photothermal agent with a 56.7% photothermal conversion efficiency for photothermal therapy of cancer cells. Nanoscale 6, 3274–3282. 10.1039/c3nr06242b 24509646

[B15] Li B. Ye K. Zhang Y. Qin J. Zou R. Xu K.. (2015). Photothermal theragnosis synergistic therapy based on bimetal sulphide nanocrystals rather than nanocomposites. Adv. Mater. Weinheim. 27, 1339–1345. 10.1002/adma.201404257 25639509

[B16] Li B. Yuan F. He G. Han X. Wang X. Qin J. (2017). Ultrasmall CuCo_2_S_4_ nanocrystals: all-in-one theragnosis nanoplatform with magnetic resonance/near-infrared imaging for efficiently photothermal therapy of tumors. Adv. Funct. Mater. 27:1606218 10.1002/adfm.201606218

[B17] Li B. Zhang Y. Zou R. Wang Q. Zhang B. An L.. (2014b). Self-assembled WO_3−x_ hierarchical nanostructures for photothermal therapy with a 915 nm laser rather than the common 980 nm laser. Dalton Trans. 43, 6244–6250. 10.1039/c3dt53396d 24598863

[B18] Liu X. Wang X. Swihart M. T. (2013). Cu_2−x_S_1−y_Se_y_ alloy nanocrystals with broadly tunable near-infrared localized surface plasmon resonance. Chem. Mater. 25, 4402–4408. 10.1021/cm402848k

[B19] Luther J. M. Jain P. K. Ewers T. Alivisatos A. P. (2011). Localized surface plasmon resonances arising from free carriers in doped quantum dots. Nat. Mater. 10, 361–366. 10.1038/nmat3004 21478881

[B20] Manthiram K. Alivisatos A. P. (2012). Tunable localized surface plasmon resonances in tungsten oxide nanocrystals. J. Am. Chem. Soc. 134, 3995–3998. 10.1021/ja211363w 22332881

[B21] Roper D. K. Ahn W. Hoepfner M. (2007). Microscale heat transfer transduced by surface plasmon resonant gold nanoparticles. J. Phys. Chem. C 111, 3636–3641. 10.1021/jp064341w 19011696 PMC2583113

[B22] Scotognella F. Della Valle G. Srimath Kandada A. R. Dorfs D. Zavelani-Rossi M. Conforti M. (2011). Plasmon dynamics in colloidal Cu2-xSe nanocrystals. Nano Lett. 11, 4711–4717. 10.1021/nl202390s 21939261

[B23] Smith A. M. Mancini M. C. Nie S. M. (2009). Bioimaging second window for *in vivo* imaging. Nat Nanotech 4, 710–711. 10.1038/nnano.2009.326 19898521 PMC2862008

[B24] Tang H. Shen S. Guo J. Chang B. Jiang X. Yang W. (2012). Gold nanorods@mSiO_2_ with a smart polymer shell responsive to heat/near-infrared light for chemo-photothermal therapy. J. Mater. Chem. 22:16095 10.1039/c2jm32599c

[B25] Tian Q. Hu J. Zhu Y. Zou R. Chen Z. Yang S. (2013). Sub-10 nm Fe_3_O_4_@Cu_2−x_S core-shell nanoparticles for dual-modal imaging and photothermal therapy. J. Am. Chem. Soc. 135, 8571–8577. 10.1021/ja4013497 23687972

[B26] Tian Q. Jiang F. Zou R. Liu Q. Chen Z. Zhu M.. (2011). Hydrophilic Cu_9_S_5_ nanocrystals: a photothermal agent with a 25.7% heat conversion efficiency for photothermal ablation of cancer cells *in vivo*. ACS Nano 5, 9761–9771. 10.1021/nn203293t 22059851

[B27] Wang J. J. Xue D. J. Guo Y. G. Hu J. S. Wan L. J. (2011). Bandgap engineering of monodispersed Cu_2−x_S_*y*_Se_1−y_ nanocrystals through chalcogen ratio and crystal structure. J. Am. Chem. Soc. 133, 18558–18561. 10.1021/ja208043g 22023550

[B28] Wang X. Swihart M. T. (2015). Controlling the size, shape, phase, band gap, and localized surface plasmon resonance of Cu_2−x_S and Cu_*x*_In_*y*_S nanocrystals. Chem. Mater. 27, 1786–1791. 10.1021/cm504626u

[B29] Wu S. Butt H. J. (2016). Near-infrared-sensitive materials based on upconverting nanoparticles. Adv. Mater. Weinheim. 28, 1208–1226. 10.1002/adma.201502843 26389516

[B30] Yang C. Ma L. Zou X. Xiang G. Chen W. (2013). Surface plasmon-enhanced Ag/CuS nanocomposites for cancer treatment. Cancer Nanotechnol. 4, 81–89. 10.1007/s12645-013-0039-2 26069503 PMC4452078

[B31] Yavuz M. S. Cheng Y. Chen J. Cobley C. M. Zhang Q. Rycenga M.. (2009). Gold nanocages covered by smart polymers for controlled release with near-infrared light. Nat. Mater. 8, 935–939. 10.1038/nmat2564 19881498 PMC2787748

[B32] Zhao Y. Pan H. Lou Y. Qiu X. Zhu J. Burda C. (2009). Plasmonic Cu(2-x)S nanocrystals: optical and structural properties of copper-deficient copper(I) sulfides. J. Am. Chem. Soc. 131, 4253–4261. 10.1021/ja805655b 19267472

