# Supplementary material for: Cu5FeS4 Nanoparticles With Tunable Plasmon Resonances for Efficient Photothermal Therapy of Cancers
Source: Front Bioeng Biotechnol. 2020 Feb 18;8:21. doi: 10.3389/fbioe.2020.00021 (PMC7039924; doi:10.3389/fbioe.2020.00021)
Supplement: Supplementary file 1 [file Data_Sheet_1.docx]

**Supporting Information**

Cu_5_FeS_4_ Nanoparticles with Tunable Plasmon Resonances for Efficient Photothermal Therapy of Cancers

*Lei Yuan^1^, Weiwei Hu^2^, Hui Zhang^1^, Long Chen^1^, Jianyu Wang^1^, Qiang Wang^1^**

*^1^Xuzhou Cancer Hospital, Xuzhou, China*

*^2^TheAffiliated Hospital of Xuzhou Medical University, Xuzhou, China*

*Qiang Wang, Email: doctorwang618@126.com*


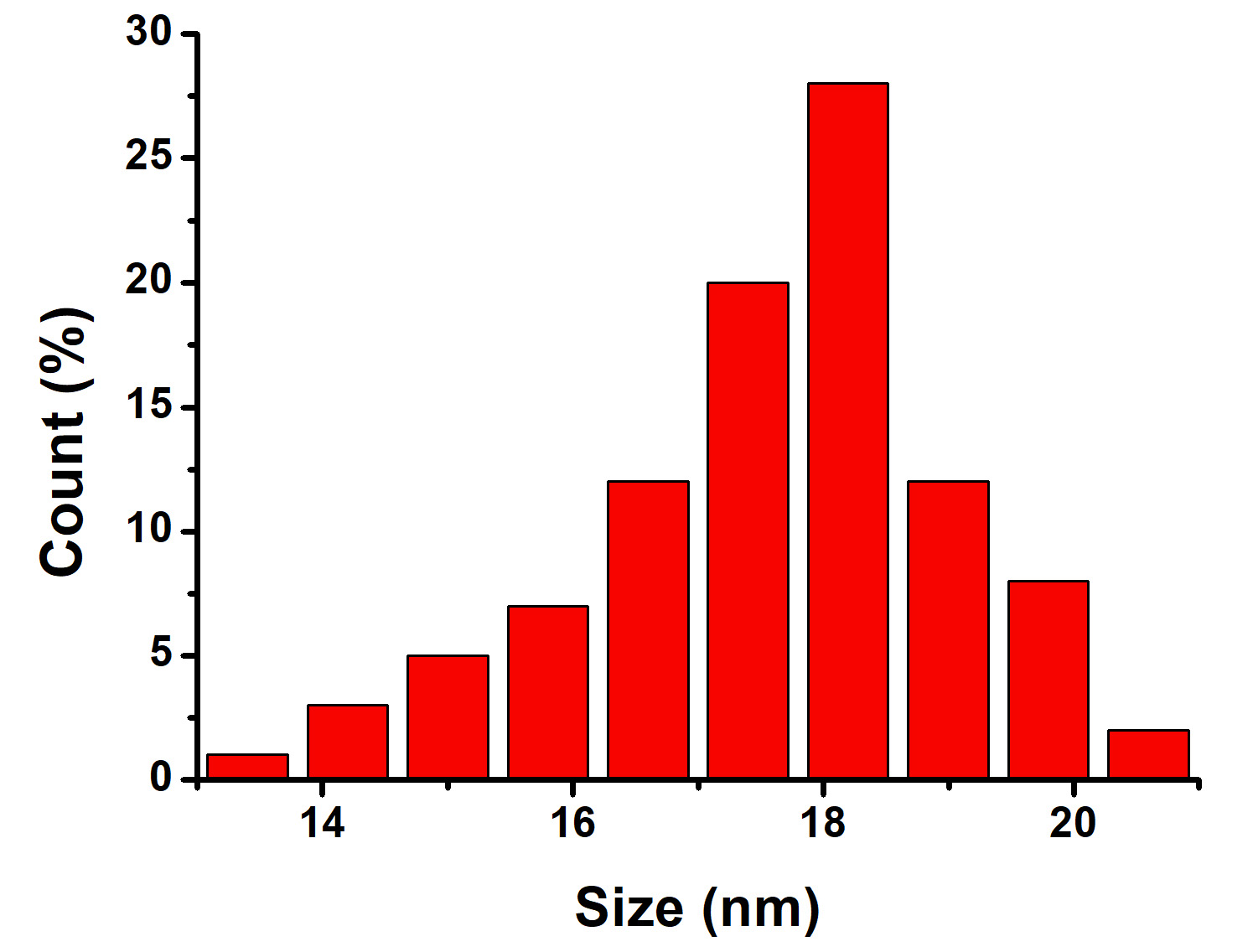


Figure S1. Size distribution of the CFS nanoparticles.


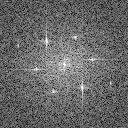


Figure S2. Selected area electron diffraction of the individual CFS nanoparticles.


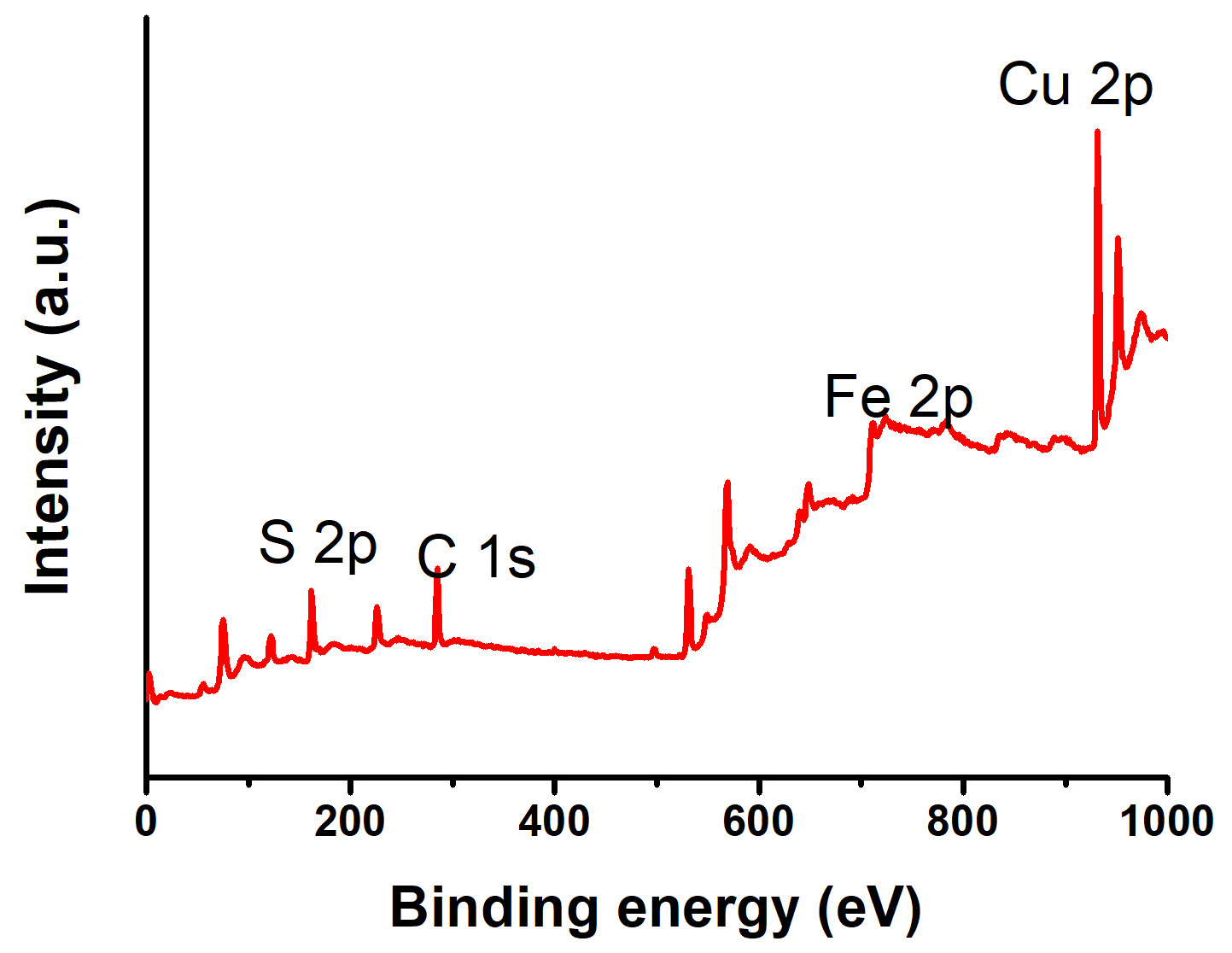


Figure S3. XPS spectrum of CFS nanoparticles.


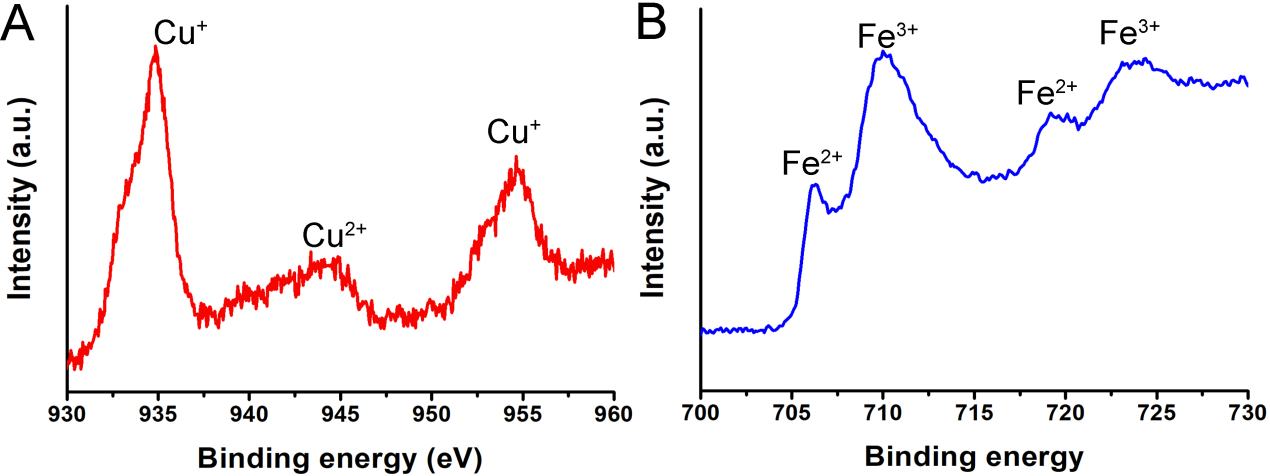


Figure S4. The high resolution XPS spectra of (A) Fe 2p and (B) Cu 2p in CFS nanoparticles.


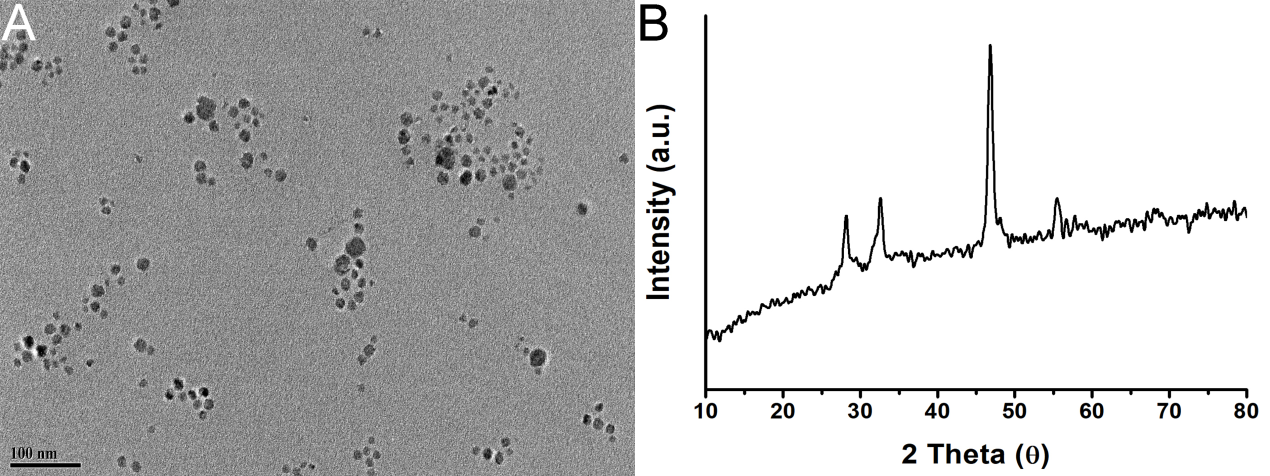


Figure S5. (A) TEM image and (B) XRD pattern of CFS nanoparticles after treatment by 20 mM DDPA.


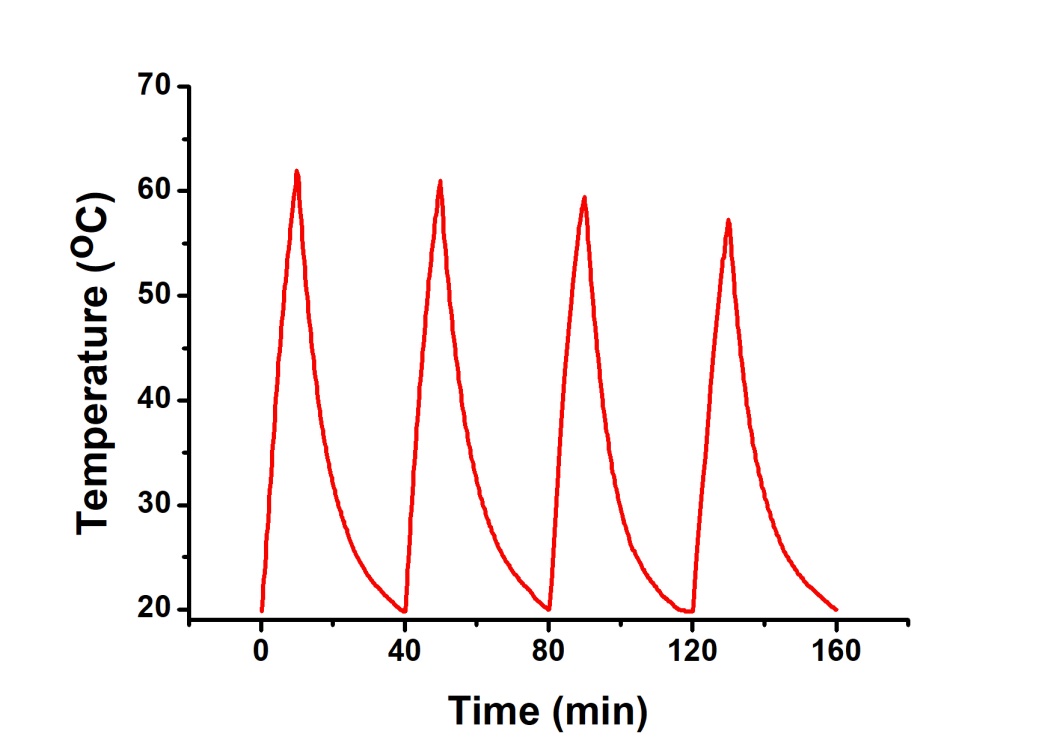


Figure S6. Temperature elevation of CFS nanoparticles over four LASER ON/OFF cycles of NIR laser irradiation.


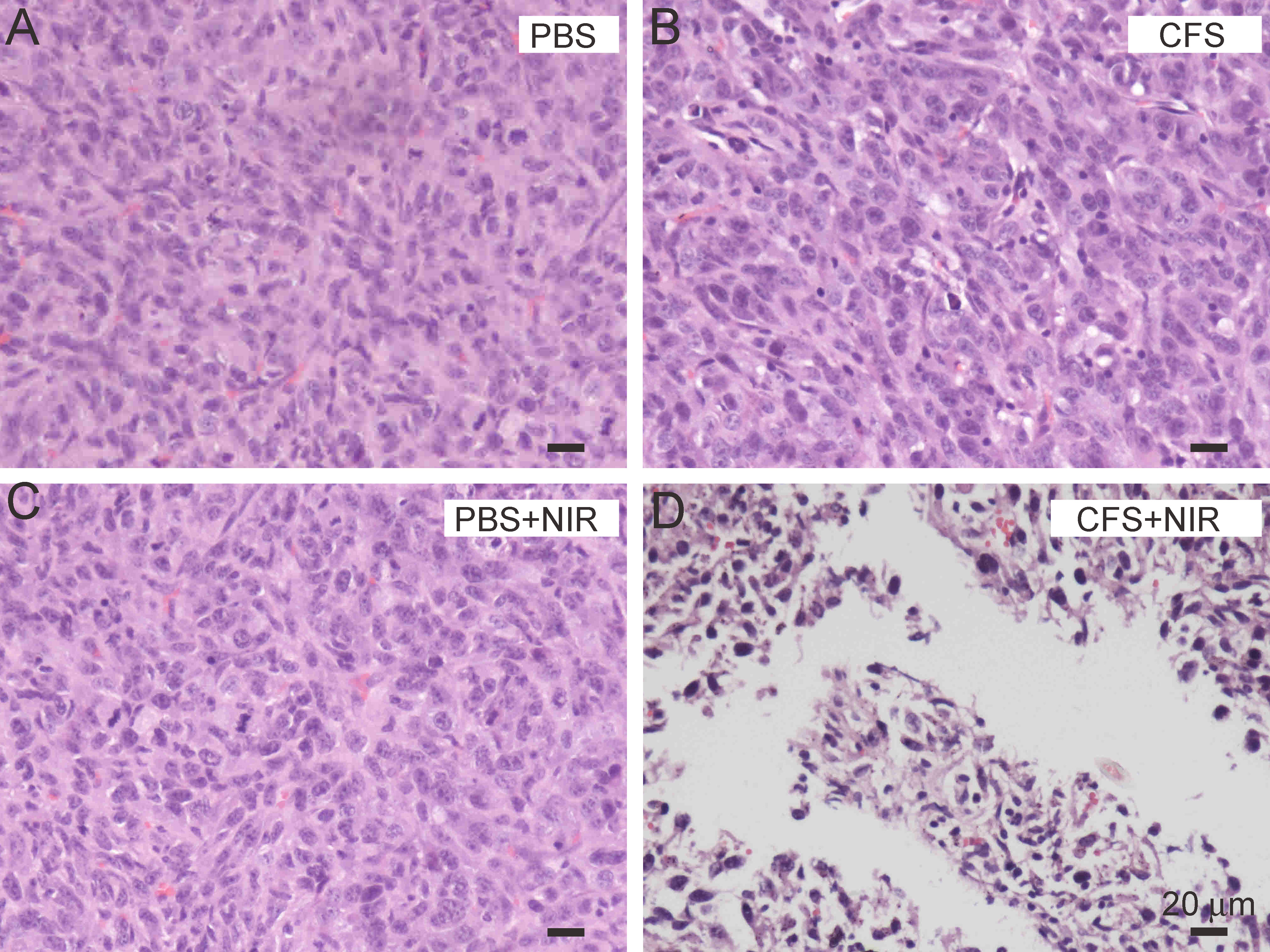


Figure S7. Representative H&E stained histological images of the tumor sections after the indicated treatments.
